# Publisher Correction: Hypermethylation of gene body CpG islands predicts high dosage of functional oncogenes in liver cancer

**DOI:** 10.1038/s41467-018-06482-w

**Published:** 2018-09-25

**Authors:** Maria Arechederra, Fabrice Daian, Annie Yim, Sehrish K. Bazai, Sylvie Richelme, Rosanna Dono, Andrew J. Saurin, Bianca H. Habermann, Flavio Maina

**Affiliations:** 1 Aix Marseille Univ, CNRS, Developmental Biology Institute of Marseille (IBDM), Parc Scientifique de Luminy, Aix Marseille Univ, 13009 Marseille, France; 2 Computational Biology Group, Max Planck Institute of Biochemistry, 82152 Martinsried, Germany

Correction to: *Nature Communications*; 10.1038/s41467-018-05550-5; published online 08 August 2018

In the original version of this Article, the sixth sentence of the abstract incorrectly read ‘Most of the genes upregulated and with hypermethylated CGIs in the Alb-R26Met HCC model undergo the same change’, and should have read ‘Most of the genes upregulated and with hypermethylated CGIs in the Alb-R26Met HCC model undergo the same change in a large proportion of HCC patients’. This has been corrected in both the PDF and HTML versions of the Article.

