# Supplementary material for: Hypermethylation of gene body CpG islands predicts high dosage of functional oncogenes in liver cancer
Source: Nat Commun. 2018 Aug 8;9:3164. doi: 10.1038/s41467-018-05550-5 (PMC6082886; doi:10.1038/s41467-018-05550-5)
Supplement: Supplementary file 1 — Supplementary Information [file 41467_2018_5550_MOESM1_ESM.pdf]

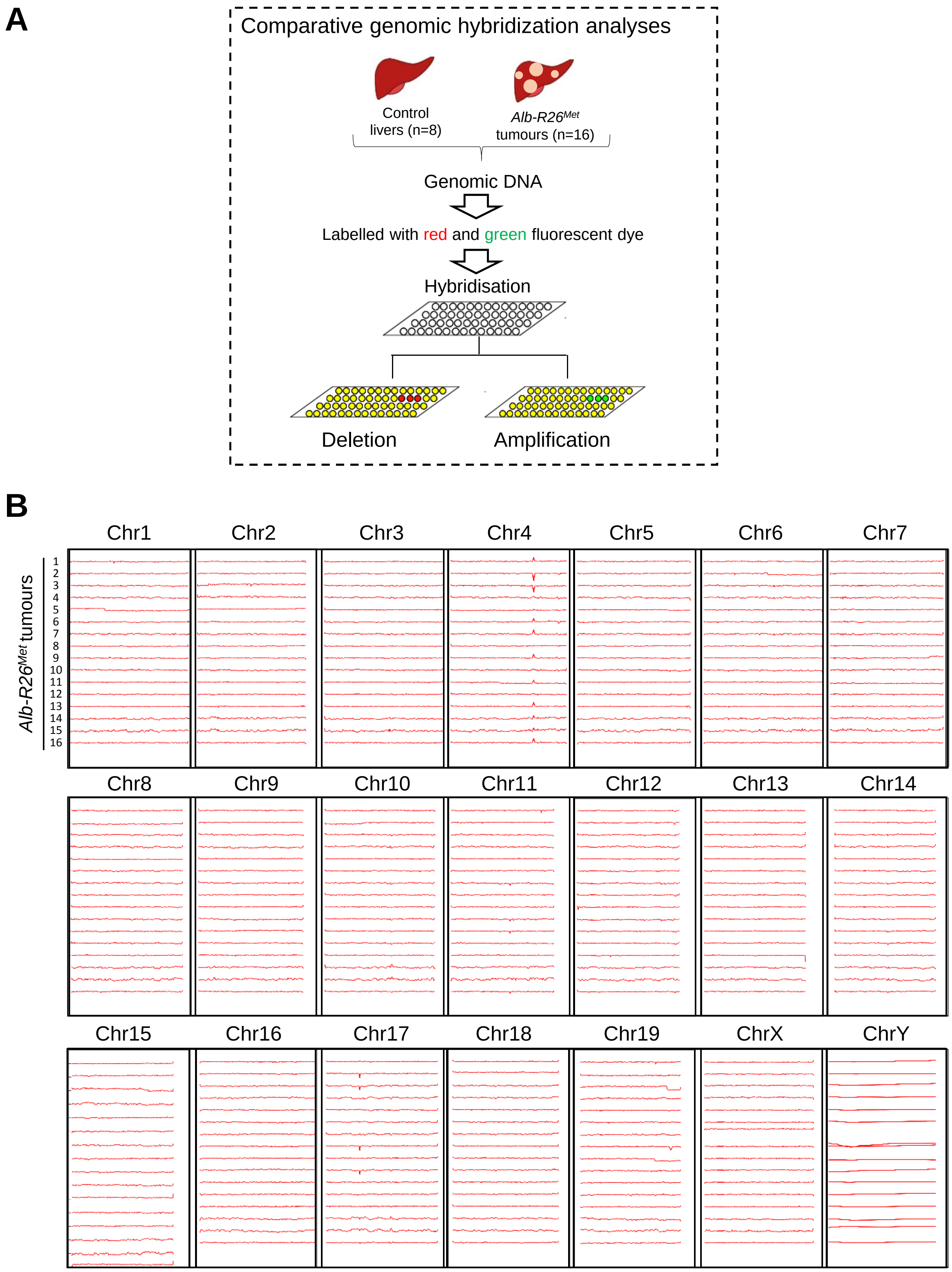

Supplementary Figure 1. Tumorigenesis modelled by the *Alb-R26^{Met}* mice is characterized by a stable chromosome context. (A) Schematic representation of the experimental setting used to analyse genomic DNA from *Alb-R26^{Met}* HCC (n=16) and control livers (n=8) through Comparative Genomic Hybridization (CGH) Microarray. (B) Overall picture of the CGH results in the 19 autosomal and the 2 sex chromosome pairs of the analysed *Alb-R26^{Met}* tumours. Note that tumours develop in a stable chromosome context; only 2 regions in chromosome 4 and chromosome 17 showed some variations in a proportion of analysed tumours.

A

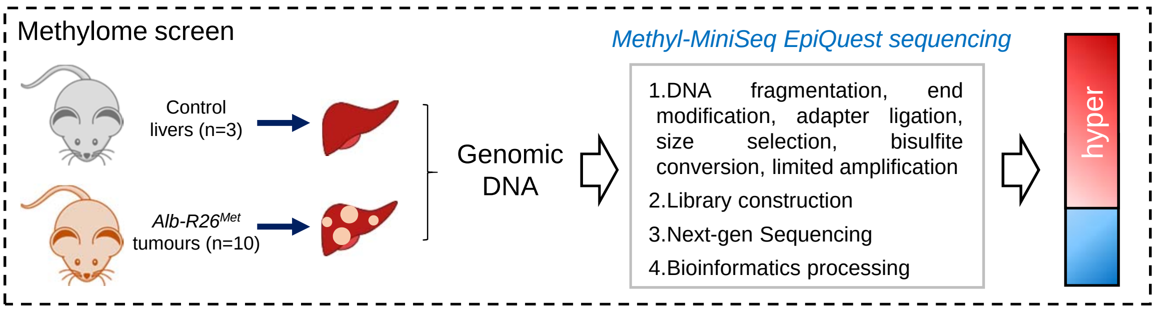

B

C

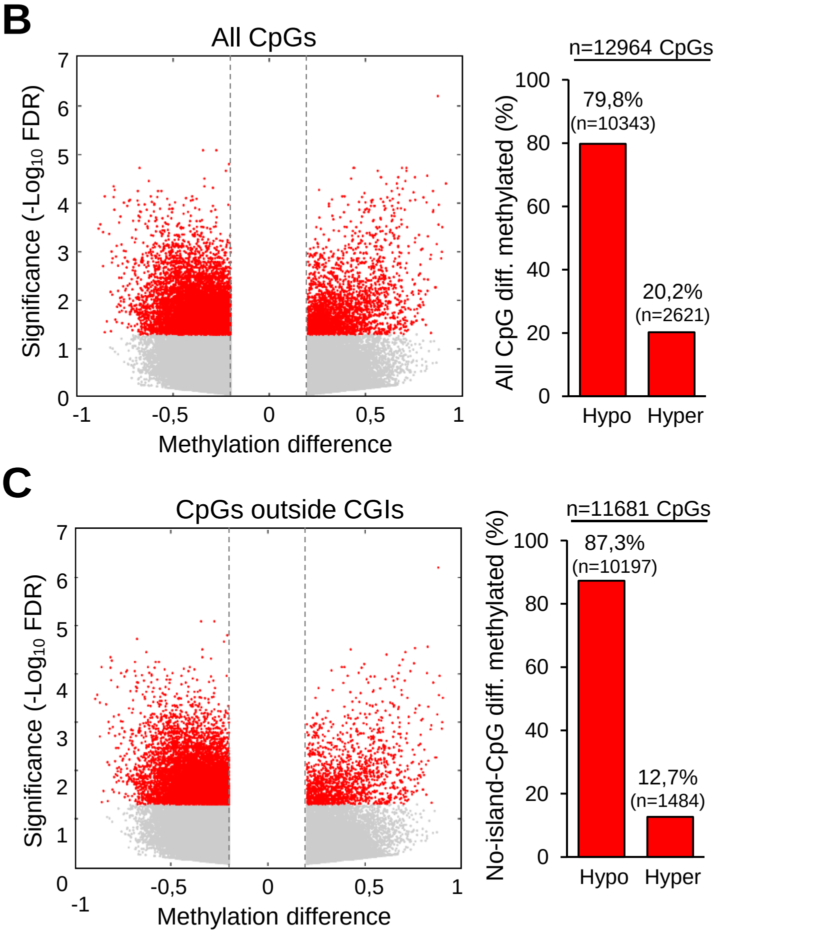

E

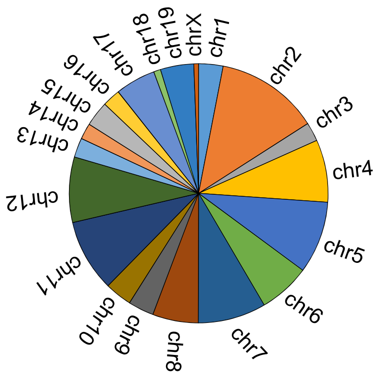

	#CpGs DM
chr1	35
chr2	149
chr3	27
chr4	90
chr5	104
chr6	74
chr7	98
chr8	66
chr9	37
chr10	39
chr11	104
chr12	94
chr13	28
chr14	23
chr15	36
chr16	26
chr17	58
chr18	10
chr19	48
chrX	7
Total	1153

D

	Number			Percentage	
	**Total**	**Hyper**	**Hypo**	**Hyper**	**Hypo**
**CpGs**	1153	1018	135	88,291414	11,708586
**CGI**	513	420	94	81,871345	18,323587
**genes**	431	348	86	80,742459	19,953596

Supplementary Figure 2. Focal CGI hypermethylation and widespread CpG hypomethylation in *Alb-R26^{Met}* tumours. (A) Schematic representation of experimental settings employed for the methylome screen. Genomic DNA from *Alb-R26^{Met}* HCC (n=10) and controls (n=3) was used to examine the DNA methylation status of a CGI-enriched fraction and raw data were used for bioinformatics processing. (B and C) Volcano plot reporting methylation differences with significance (expressed as -Log$_{10}$ FDR) for all measured CpGs (B) and CpGs located outside CGIs (C) in *Alb-R26^{Met}* tumours versus control (left). Significant differences (methylation difference>0.2 and FDR<0.05) are shown in red. Graph reporting the percentage (and numbers) of hypomethylated versus hypermethylated CpGs (right). (D) Table reporting the number and percentage of CpGs, the corresponding CGIs and genes, differentially methylated, specifying the ones hypermethylated versus those hypomethylated. (E) Chromosomic distribution of the 1153 CpGs differentially methylated in *Alb-R26^{Met}* tumours compared to control livers.

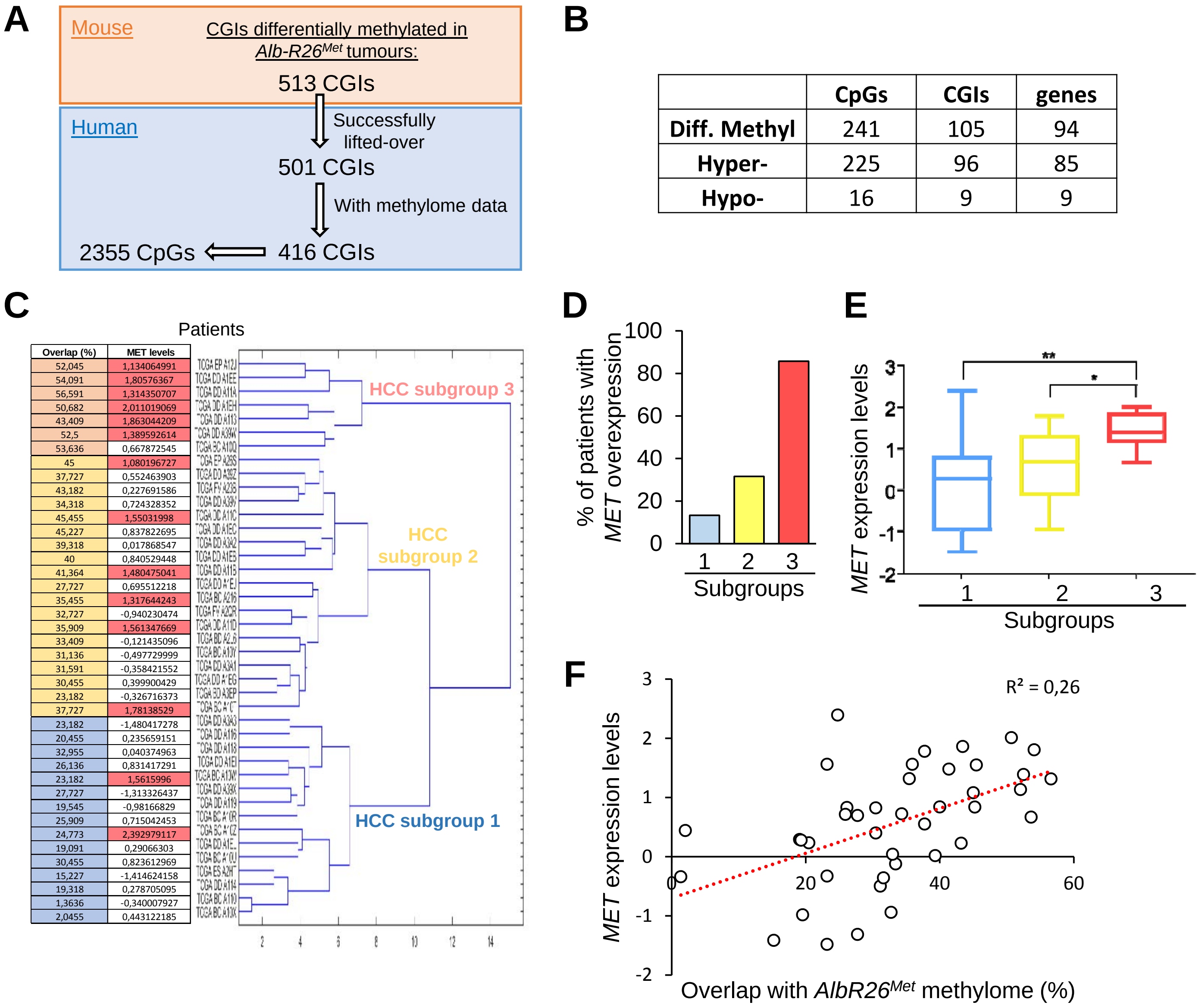

Supplementary Figure 3. Comparison of methylome outcomes of *Alb-R26^{Met}* tumours with those of the TCGA cohort (41 HCC patients). (A) Schematic representation of the mouse-human CGI lift-over performed to extract the human CGIs corresponding to the 513 mouse CGIs differentially methylated in *Alb-R26^{Met}* tumours. 501 CGIs were successfully mapped in human, and methylome data were available for 416 CGIs. (B) Table reporting the number and percentage of CpGs, the corresponding CGIs and genes, differentially methylated, specifying the ones hypermethylated versus those hypomethylated. Data refer to those reported in Figure 1E. (C) Hierarchical clustering of HCC patients based on the 416 CGIs differentially methylated in *Alb-R26^{Met}* tumours. Data refer to those reported in Figure 1F, specifying the patient ID, the percentage overlap, and the *MET* expression levels. (D) Graph showing the percentage of patients with *MET* overexpression in the three HCC subgroups reported in Figure 1F. Note that *MET* is overexpressed in 86% (6/7) of HCC patients belonging to the HCC subgroup 3 (the subgroup that best overlap with CGI methylation changes in *Alb-R26^{Met}*), 32% (6/19) to the HCC subgroup 2, and only 13% (2/15) to the HCC subgroup 1. (E) Graph reporting *MET* expression levels in the three HCC subgroups reported in Figure 1F. (F) For the 41 HCC patients, correlation between *MET* expression levels and percentage of overlap with the CGIs found differentially methylated in the *Alb-R26^{Met}*genetic setting.

A

	CpGs	CGIs	genes
**Diff. Methyl**	269	110	100
**Hyper-**	255	103	93
**Hypo-**	14	7	7

B

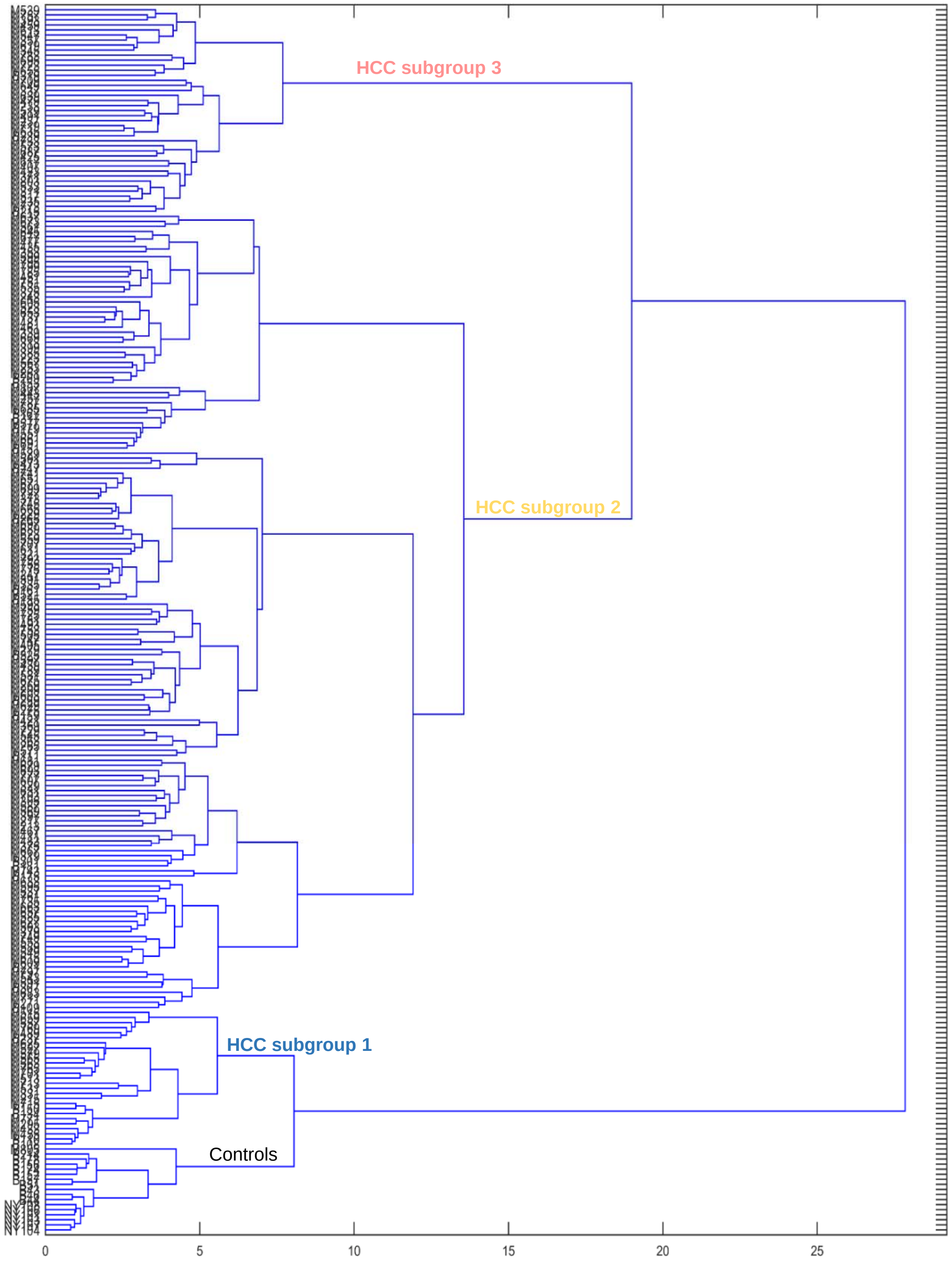

Supplementary Figure 4. Comparison of methylome outcomes of *Alb-R26*^{Met} tumours with those of a cohort of 234 human samples (from GSE56588; 224 HCC patients and 10 control individuals). (A) Table reporting the number and percentage of CpGs, the corresponding CGIs and genes, differentially methylated, specifying the ones hypermethylated versus those hypomethylated. Data refer to those reported in Figure 1G. (B) Hierarchical clustering of HCC patients based on the 416 CGIs differentially methylated in *Alb-R26*^{Met} tumours. Data refer to those reported in Figure 1H.

Pathway enrichment analysis: genes differentially methylated and differentially expressed in *Alb-R26^{Met}* tumours

Pathway	p value
MAPK signaling pathway - Homo sapiens (human)	0.00015777
HTLV-I infection - Homo sapiens (human)	0.00016107
Axon guidance - Homo sapiens (human)	0.0002355
Nicotine addiction - Homo sapiens (human)	0.00028279
Viral carcinogenesis - Homo sapiens (human)	0.00043804
Pathways in cancer - Homo sapiens (human)	0.0014551
Neuroactive ligand-receptor interaction - Homo sapiens (human)	0.0017757
TGF-beta signaling pathway - Homo sapiens (human)	0.0023777
GABAergic synapse - Homo sapiens (human)	0.0027043
Morphine addiction - Homo sapiens (human)	0.002966
Cell cycle - Homo sapiens (human)	0.0068301
Osteoclast differentiation - Homo sapiens (human)	0.007425
Type II diabetes mellitus - Homo sapiens (human)	0.0092091
Tight junction - Homo sapiens (human)	0.015343
Renal cell carcinoma - Homo sapiens (human)	0.017431
Calcium signaling pathway - Homo sapiens (human)	0.018154
Epithelial cell signaling in Helicobacter pylori infection - Homo sapiens (human)	0.01891
Melanoma - Homo sapiens (human)	0.019414
Adherens junction - Homo sapiens (human)	0.020952
cAMP signaling pathway - Homo sapiens (human)	0.022263
Focal adhesion - Homo sapiens (human)	0.022533
Epstein-Barr virus infection - Homo sapiens (human)	0.023628
Rap1 signaling pathway - Homo sapiens (human)	0.025603
Protein digestion and absorption - Homo sapiens (human)	0.031017
NF-kappa B signaling pathway - Homo sapiens (human)	0.034037
Endocrine resistance - Homo sapiens (human)	0.034652
Amoebiasis - Homo sapiens (human)	0.034652
Estrogen signaling pathway - Homo sapiens (human)	0.035891
Chagas disease (American trypanosomiasis) - Homo sapiens (human)	0.038408
Leukocyte transendothelial migration - Homo sapiens (human)	0.044908
Cholinergic synapse - Homo sapiens (human)	0.044908
Cytokine-cytokine receptor interaction - Homo sapiens (human)	0.045221
Serotonergic synapse - Homo sapiens (human)	0.045573

Supplementary Figure 5. KEGG pathway enrichment analysis for genes with changes in CGI methylation and expression in *Alb-R26^{Met}* tumours, ranked according to their p-value. Data reported in Figure 3A are in pink.

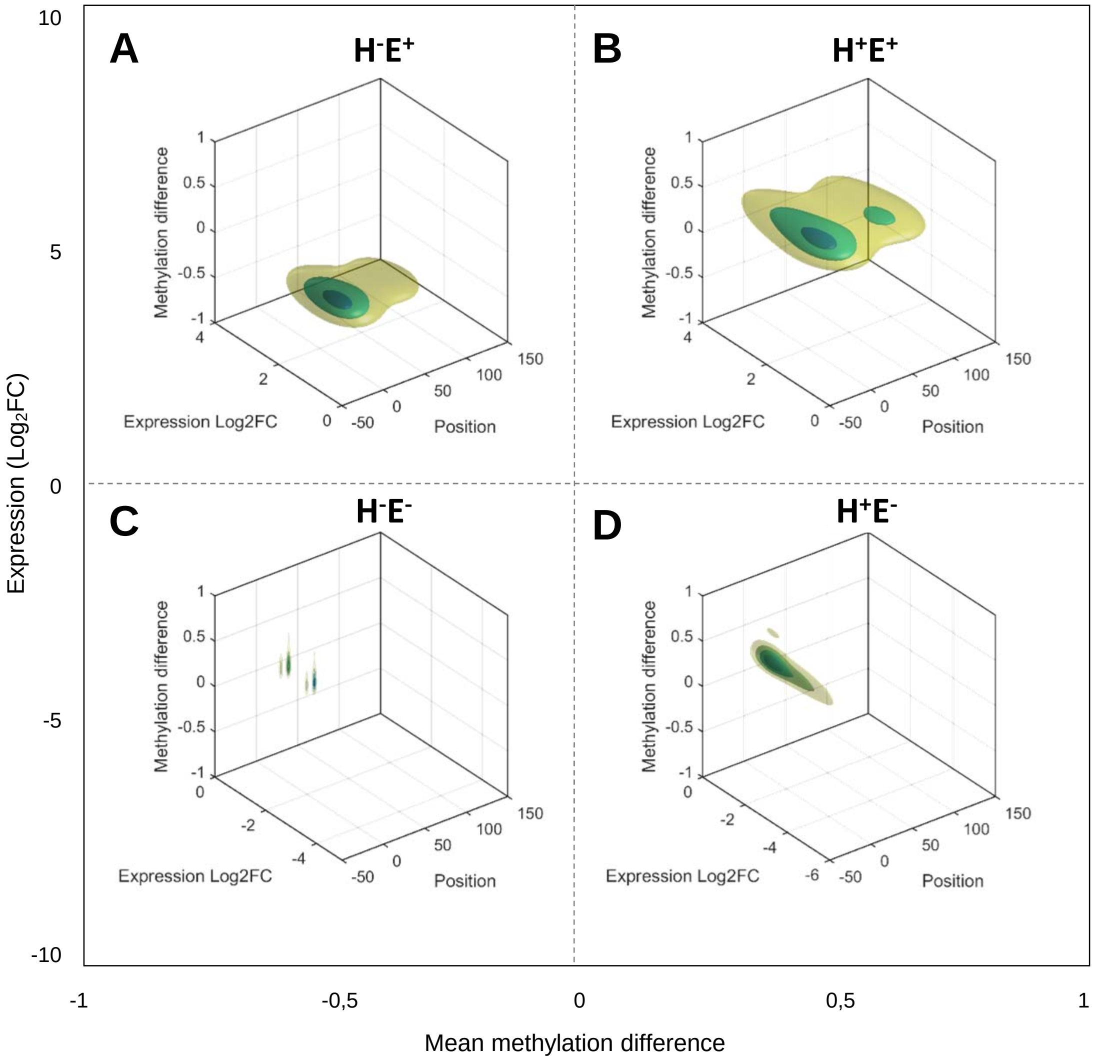

Supplementary Figure 6. 3D density plots showing the distribution of genes according to their relative position to the ATG (as percentage), gene expression level (as Log_2 FC), and CGI methylation difference (as β-value). Genes: overexpressed with hypomethylated CGI (A); overexpressed with hypermethylated CGI (B); downregulated with hypomethylated CGI (C); downregulated with hypermethylated CGI (D).

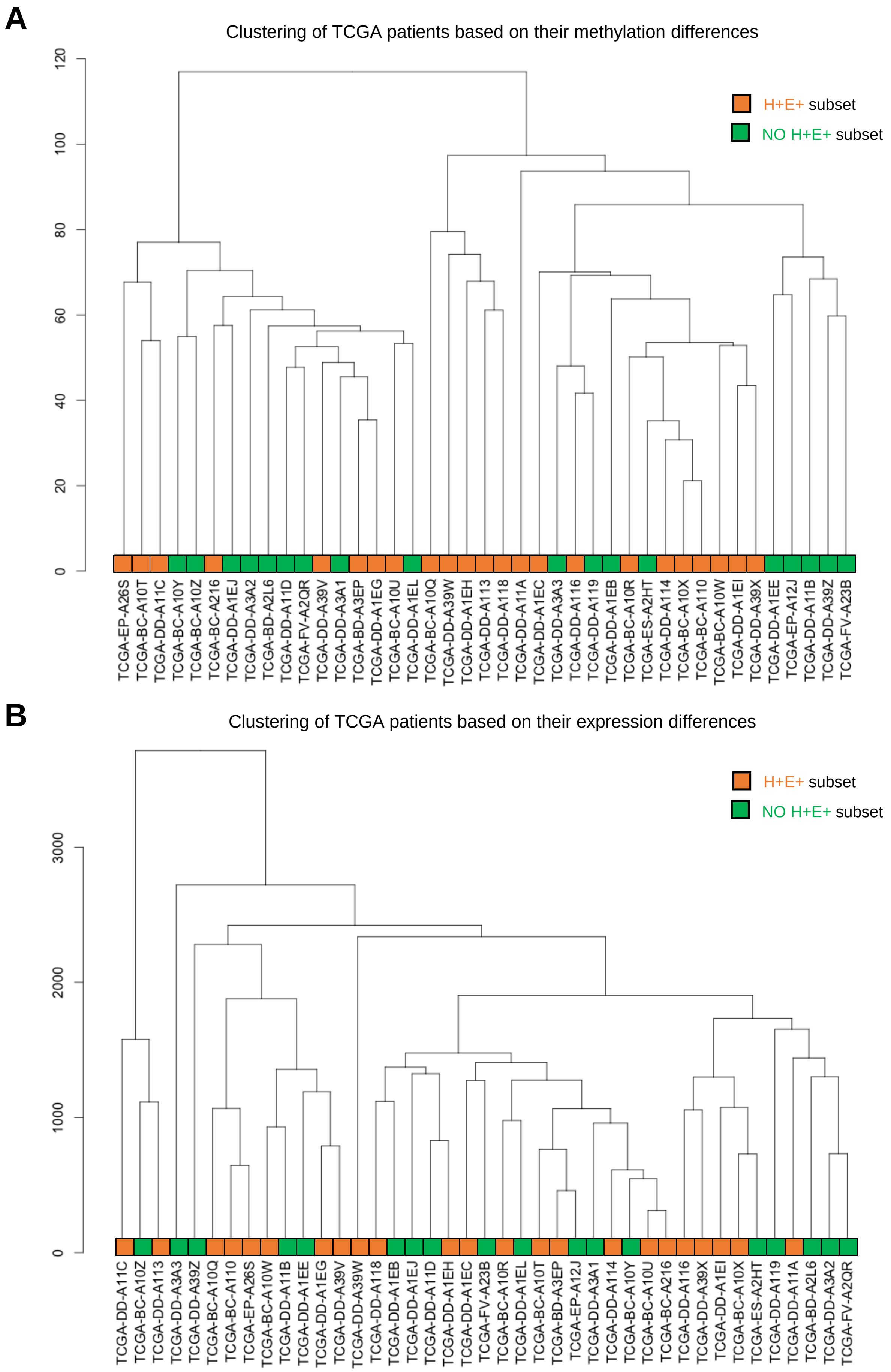

Supplementary Figure 7. Hierarchical clustering analysis of the TCGA cohort (41 HCC patients) based on either global DNA methylome (A) or transcriptome (B) outcomes. In orange: H$^+$E$^+$ patient subset. In green: “NO H$^+$E$^+$” patient subset.

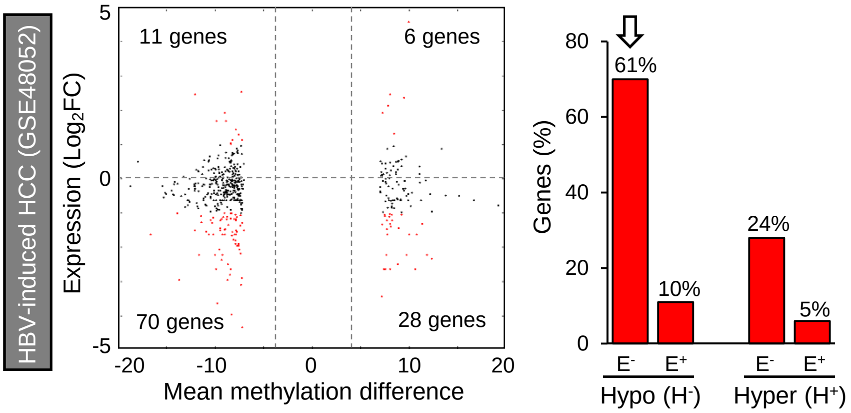

Supplementary Figure 8. Mouse *HBx*^{tg} tumours are characterized by an enrichment in genes downregulated and with hypomethylated CGIs. Left: Methylation differences versus expression for all genes with CGIs hypermethylated (H^+) or hypomethylated (H^-) in *HBx*^{gt} tumours. Expression values are relative to controls. Dots correspond to single differentially methylated CpG and the corresponding gene expression (genes which expression is significantly below or above Log_2 fold change (FC) ±1 are indicated in red). Right: Graph reporting the percentage of downregulated (E^-) and upregulated (E^+) genes among those with a hypomethylated (H^-) or hypermethylated (H^+) CGI. Note the enrichment of genes downregulated and with hypomethylated CGIs in *HBx*^{gt} tumours (indicated by an arrow), in contrast to the enrichment of gene overexpressed and with hypermethylated CGIs in *Alb-R26*^{Met} tumours.

Subset	Patients	Alcohol	NAFLD	HepatitisB	HepatitisC	Hemochromatosis	TOTAL
$H^{+}E^{+}$ patient subset	TCGA-BC-A10Q	0	0	0	0	0	0
	TCGA-DD-A1EH	0	0	1	0	0	1
	TCGA-DD-A1EC	0	0	0	0	0	0
	TCGA-DD-A113	0	0	0	0	0	0
	TCGA-DD-A11A	0	0	0	0	0	0
	TCGA-DD-A39W	0	0	0	0	0	0
	TCGA-BC-A216	0	0	0	0	0	0
	TCGA-EP-A26S	1	0	0	0	0	1
	TCGA-DD-A11C	0	0	0	0	0	0
	TCGA-BC-A10T	0	0	0	0	0	0
	TCGA-DD-A118	0	0	0	0	0	0
	TCGA-DD-A39X	0	0	0	0	0	0
	TCGA-DD-A1EI	0	0	1	0	0	1
	TCGA-BC-A10R	0	0	0	0	0	0
	TCGA-BC-A10W	0	0	1	0	0	1
	TCGA-BC-A10U	1	0	0	0	0	1
	TCGA-DD-A39V	0	0	0	0	0	0
	TCGA-BD-A3EP	0	0	0	0	0	0
	TCGA-DD-A1EG	1	1	0	0	0	2
	TCGA-DD-A114	0	0	0	1	0	1
	TCGA-DD-A116	0	0	1	0	0	1
	TCGA-BC-A110	1	0	0	0	0	1
	TCGA-BC-A10X	0	0	0	0	0	0
NO H+E+ patient subset	TCGA-BD-A2L6	1	0	0	0	0	1
	TCGA-BC-A10Z	0	0	0	0	0	0
	TCGA-BC-A10Y	1	0	0	0	0	1
	TCGA-FV-A2QR	0	0	0	0	0	0
	TCGA-DD-A1EJ	0	0	0	0	0	0
	TCGA-DD-A1EL	0	0	1	0	0	1
	TCGA-DD-A11D	0	1	0	0	0	1
	TCGA-DD-A3A1	0	0	0	0	0	0
	TCGA-DD-A1EE	0	0	0	1	0	1
	TCGA-EP-A12J	0	0	0	1	0	1
	TCGA-FV-A23B	0	0	0	1	0	1
	TCGA-DD-A11B	0	1	0	0	0	1
	TCGA-DD-A3A3	0	0	0	0	0	0
	TCGA-DD-A1EB	0	0	0	0	0	0
	TCGA-DD-A3A2	0	0	0	0	0	0
	TCGA-DD-A39Z	1	0	0	0	0	1
	TCGA-DD-A119	0	0	1	0	0	1
	TCGA-ES-A2HT	0	0	0	1	0	1

Fisher's exact test
p-value=0,2146

Supplementary Figure 9. $H^{+}E^{+}$ patients are not characterised by any specific risk factors, while show a trend of better prognosis. Table reporting risk factors associated to individual HCC patients. The presence of a risk factor is indicated as 1, whereas the absence by 0. No significant differences were found between $H^{+}E^{+}$ versus "NO $H^{+}E^{+}$" patient subsets.

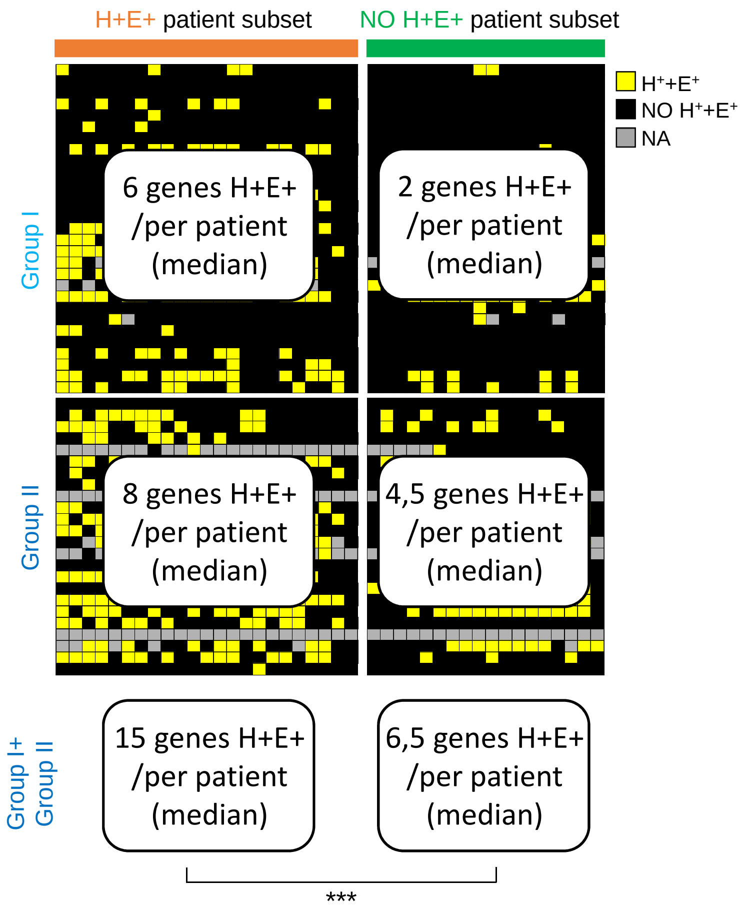

Supplementary Figure 10. Among the 55 genes identified in *Alb-R26^{Met}* tumours, a significant higher number of them is both hypermethylated and overexpressed in the H^{+}E^{+} patient subset compared to the "NO H^{+}E^{+}" subset. Heat-map showed in Figure 6D in which the number of genes both hypermethylated and overexpressed per patient (median) is reported for each subgroup. Significance is indicated on the bottom. ***: P<0.001.

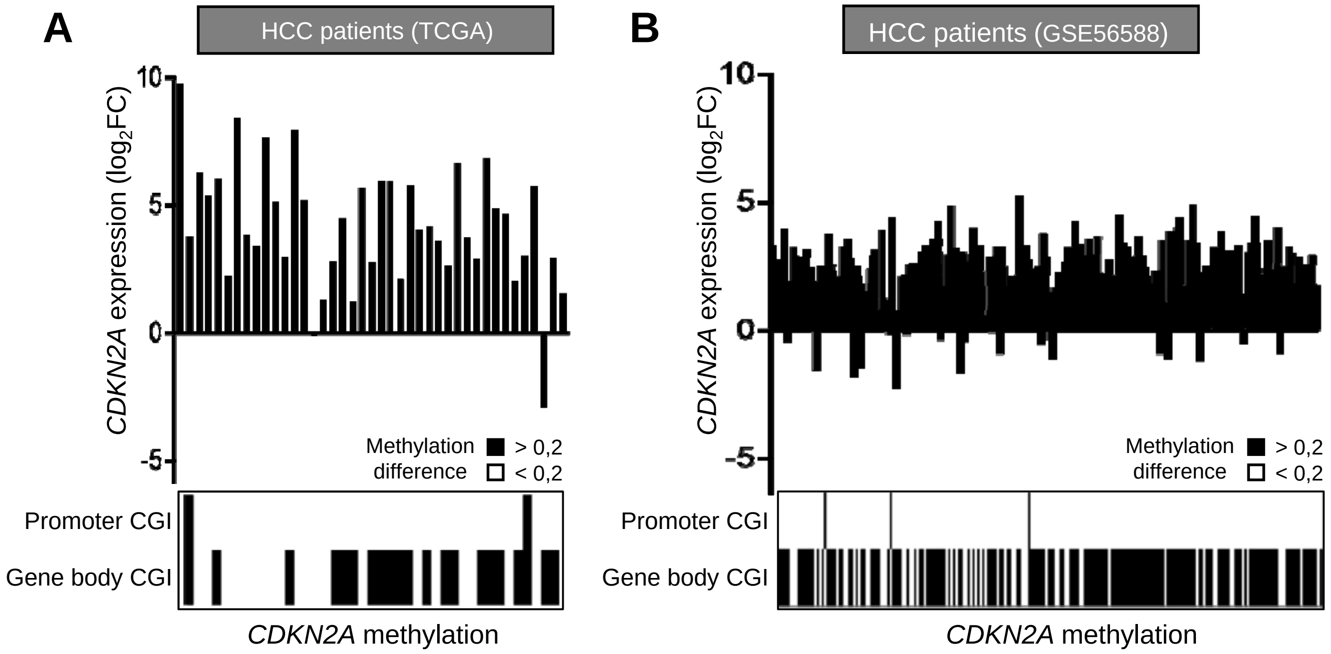

Supplementary Figure 11. The *CDKN2A* gene is overexpressed and with hypermethylated gene body CGI in the majority of HCC patients. Top: graphs reporting the expression levels of *CDKN2A* in HCC patients from the TCGA (A) and GSE56588 (B) cohorts. Bottom: schemes reporting a black line when the *CDKN2A* promoter or gene body CGI is hypermethylated (methylation difference >0.2) in HCC patients from the TCGA (A) and GSE56588 (B) cohorts. Notably, in both cohorts the majority of HCC patients carry an overexpression of *CDKN2A* (39/41 and 166/204, in the respective cohorts), which is associated with a hypermethylation of the gene body CGI (21/39 and 163/166, in the respective cohorts). In contrast, not methylation changes are detected in the promoter CGI for both HCC cohorts.

Pathway enrichment analysis: overexpressed genes with hypermethylated CGI in *Alb-R26^Met^* tumours

Pathway	p value
MAPK signaling pathway - Homo sapiens (human)	8.2428e-06
Viral carcinogenesis - Homo sapiens (human)	3.7666e-05
HTLV-I infection - Homo sapiens (human)	0.000116
Pathways in cancer - Homo sapiens (human)	0.00080777
Cell cycle - Homo sapiens (human)	0.0016141
Osteoclast differentiation - Homo sapiens (human)	0.001764
Nicotine addiction - Homo sapiens (human)	0.0025759
Tight junction - Homo sapiens (human)	0.0038538
Axon guidance - Homo sapiens (human)	0.0041683
cAMP signaling pathway - Homo sapiens (human)	0.0058036
Focal adhesion - Homo sapiens (human)	0.0058818
Epstein-Barr virus infection - Homo sapiens (human)	0.0062006
Renal cell carcinoma - Homo sapiens (human)	0.0065617
Epithelial cell signaling in Helicobacter pylori infection - Homo sapiens (human)	0.0071469
Melanoma - Homo sapiens (human)	0.0073467
Adherens junction - Homo sapiens (human)	0.0079605
TGF-beta signaling pathway - Homo sapiens (human)	0.010621
GABAergic synapse - Homo sapiens (human)	0.011577
Morphine addiction - Homo sapiens (human)	0.012317
NF-kappa B signaling pathway - Homo sapiens (human)	0.013332
Endocrine resistance - Homo sapiens (human)	0.01359
Estrogen signaling pathway - Homo sapiens (human)	0.014114
Leukocyte transendothelial migration - Homo sapiens (human)	0.017989
Cholinergic synapse - Homo sapiens (human)	0.017989
Wnt signaling pathway - Homo sapiens (human)	0.027757
Breast cancer - Homo sapiens (human)	0.028096
Maturity onset diabetes of the young - Homo sapiens (human)	0.047146

Supplementary Figure 12. KEGG pathway enrichment analysis for overexpressed genes with hypermethylated CGI in *Alb-R26^Met^* tumours, ranked according to their p-value. Data reported in Figure 6F are in blue.

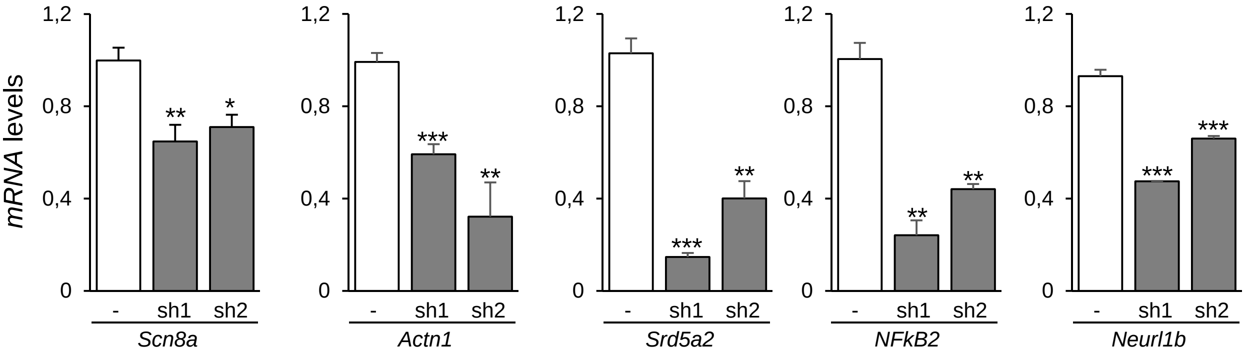

Supplementary Figure 13. Expression levels of *Scn8a*, *Actn1*, *Srd5a*, *NFkB2*, and *Neurl1b* in *Alb-R26^{Met}* HCC cells transfected with two different shRNA targeting sequences versus controls. After molecular validation, cells were used for in vitro studies reported in Figure 7.

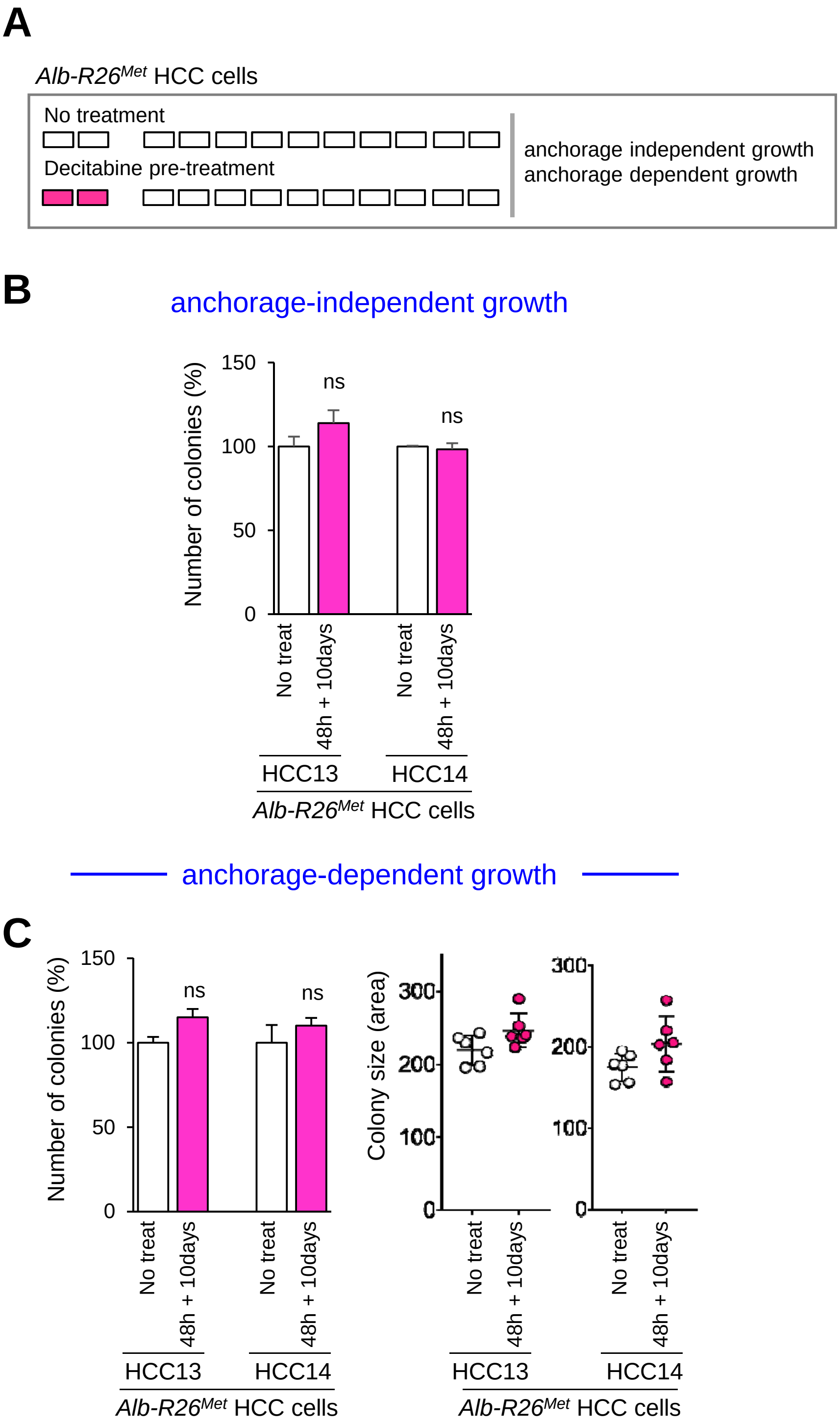

Supplementary Figure 14. Global CGI hypermethylation is functionally relevant for *Alb-R26^{Met}* tumorigenesis. (A) Scheme reporting demethylating treatment (Decitabine; 0.3µM) used for in vitro experiments with *Alb-R26^{Met}* HCC cells. Cells were pre-treated (48 h) with Decitabine, then cultured for 10 days without any treatment before using them for experiments. (B, C) Anchorage-independent (B) and anchorage-depend (C) growth assays using 2 different *Alb-R26^{Met}* HCC cell lines (HCC13 and 14) showing effects of demethylating treatments described in A. Note that HCC cells recover their tumorigenic properties when experiments are performed with cell cultured 10 days after Decitabine pre-treatment. Significant differences between groups are indicated on the top. Not significant (ns).

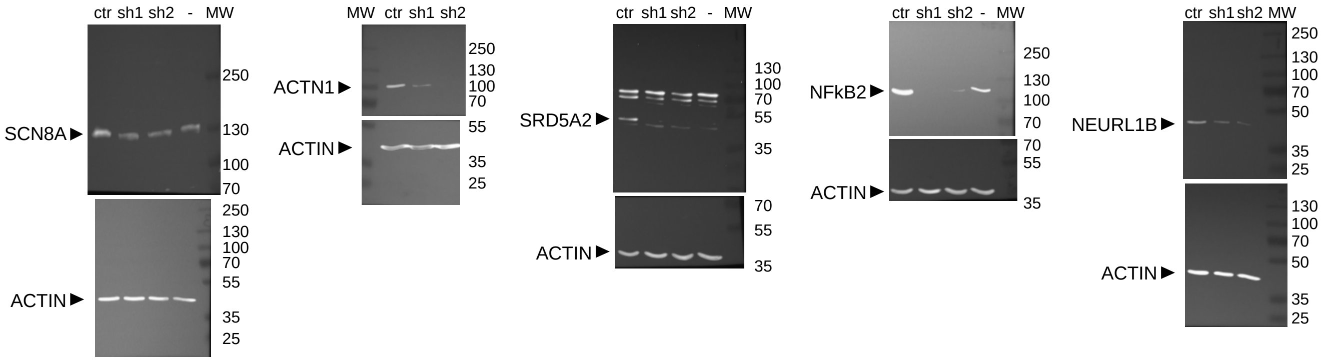

Supplementary Figure 15. Full blots of gels in which the acquisition of ECL signal performed using the MyECL imager system (without negative conversion) was merged with a picture of the membranes. The corresponding molecular weights, visible on the membranes, are indicated. These results are reported in Figure 7A.
