# Supplementary material for: Hypermethylation of gene body CpG islands predicts high dosage of functional oncogenes in liver cancer
Source: Nat Commun. 2018 Aug 8;9:3164. doi: 10.1038/s41467-018-05550-5 (PMC6082886; doi:10.1038/s41467-018-05550-5)
Supplement: Supplementary file 3 — Description of Additional Supplementary Files [file 41467_2018_5550_MOESM3_ESM.pdf]

Description of Additional Supplementary Files

File Name: Supplementary Data 1
Description: ***Alb-R26^{Met}* methylome outcomes.** For the 1153 CpG site found differentially methylated in *Alb-R26^{Met}* tumours (n=10) compared to control livers (n=3), the table reports: ID, chromosome number, CpG position (chromosome start and chromosome end), CGI genomic location, gene name (Refseq_gene), mean methylation difference (expressed as β-value), and the corresponding FDR.

File Name: Supplementary Data 2
Description: **Mouse-to-human CGI lift-over.** For comparison with human methylome data, the 513 differentially methylated CGIs in *Alb-R26^{Met}* tumours were converted from mouse (mm9) to human (hg19) using UCSC lift-over tool (standard parameters). Table reports the mouse CGI location, the gene name, and the corresponding human CGI location. Note that 501 out of 513 CGI were successfully lifted-over.

File Name: Supplementary Data 3
Description: **Expression levels of genes found differentially methylated and differentially expressed in *Alb-R26^{Met}* tumours.** Table reporting gene name, methylation status compared to control livers (red: hypermethylated; blue: hypomethylated), expression levels (expressed as log_2FC), and the corresponding FDR, for the 93 genes found differentially methylated and differentially expressed. Note that there is an enrichment of genes (55 out of 93 genes) both overexpressed and with hypermethylated CGI.

File Name: Supplementary Data 4
Description: **No methylation change at the promoter CGI of overexpressed genes with hypermethylated gene body CGI.** For the 24 genes belonging to Group II, both overexpressed and with hypermethylated CGI in gene body, unchanged methylation in promoter CGI was found. The table reports gene name, chromosomal and genomic location of the promoter CGI, and the corresponding mean methylation difference.

File Name: Supplementary Data 5
Description: **List of primer sequences used for the targeted bisulfite sequencing.** Data reported in the table are related to Figure 4.

File Name: Supplementary Data 6
Description: **Methylome and transcriptome integration analysis of HCC patients from the TCGA database.** Table related to Figure 5A. After transcriptome and methylome data integration, for each patient, the table reports numbers and percentages of: 1) total CpGs differentially methylated for genes differentially expressed; 2) hypermethylated CpGs for overexpressed genes (H^+E^+); 3) hypermethylated CpGs for downregulated genes (H^+E^-); 4) hypomethylated CpGs for overexpressed genes (H^-E^+); 5) hypomethylated CpGs for downregulated genes (H^-E^-). In addition, on the right the table reports as well for each patient to which subgroup they belong to (according to patient segregation reported in Figure 1F) and the *MET* expression levels (as Log_2FC; highlighted in red when Log_2FC is >1) . Note that patient subgroups are based on their highest percentage in one of the above 4 groups (highlighted in yellow), with 23 patients belonging to H^+E^+ subset, whereas the other 18 patients (NO H^+E^+ subset) belong to either H^+E^- or H^-E^- subsets. In each subset, patients are organized according to the total number of differentially methylated CpGs in their specific subgroup (indicated in bold).

File Name: Supplementary Data 7
Description: **Availability of methylation and expression data, related to HCC patients from the TCGA cohort, for the 55 genes identified in *Alb-R26^{Met}* tumours.** For each gene, the number of CGIs in the human gene, as well as the availability of methylation and expression data is reported.

File Name: Supplementary Data 8
Description: **The 55 genes identified in *Alb-R26^{Met}* tumours are also upregulated in H^+E^+ patient subsets.** Table related to figure 6A, reporting expression levels (as Log_2FC) of genes belonging to Group I and II (rows: organized according to the relative position to the ATG) in individual patients (columns: organized as in Figure 5A). Red: upregulated genes; green: downregulated genes. Black: not differentially expressed. White: data not available. The H^+E^+ patient subset is highlighted in orange (left), whereas all other patients in green (right).

File Name: Supplementary Data 9
Description: **The 55 genes identified in *Alb-R26^{Met}* tumours are also upregulated and with hypermethylated CGI in H^+E^+ patient subset.** Table related to figure 6A and D, reporting in yellow when the gene is both upregulated and with hypermethylated CGI in individual patients (rows: genes organized according to the relative position to the ATG; columns: patients organized as in Figure 5A). Black: genes not upregulated and with hypermethylated CGI in the corresponding HCC patient. Grey: data not available.

File Name: Supplementary Data 10
Description: **Table summarising information regarding the 55 genes found overexpressed and with hypermethylated CGI in *Alb-R26^{Met}* tumours.** For the 55 genes overexpressed and with hypermethylated CGI in *Alb-R26^{Met}* tumours compared to controls, the table reports their relative position to the ATG, expression levels (as Log_2FC), and the number of paper found in Pubmed related to cancer and HCC.

File Name: Supplementary Data 11
Description: **List of primer sequences used for RT-qPCR analyses.**

File Name: Supplementary Data 12
Description: **List of shRNA targeting sequences used to downregulate gene expression in *Alb-R26^{Met}* cells.** Data reported in the table are related to Figure 7.
