# Supplementary material for: Hypermethylation of gene body CpG islands predicts high dosage of functional oncogenes in liver cancer
Source: Nat Commun. 2018 Aug 8;9:3164. doi: 10.1038/s41467-018-05550-5 (PMC6082886; doi:10.1038/s41467-018-05550-5)
Supplement: Supplementary file 8 — Supplementary Data 5 [file 41467_2018_5550_MOESM8_ESM.docx]

**Supplementary Data 5**

| **Name of the target** | **chromosome** | **strand** | **Forward Primer Sequence** | **Reverse Primer Sequence** |
| --- | --- | --- | --- | --- |
| C2cd4c | chr10 | - | GATGATAGTATATTAGATATTAGTTTAGGTGTT | ACTCCRAACATCACAAAACC |
| Actn1 | chr12 | - | GAGGAAATTAGAATAYGGGTATTTAATATTTT | CCRTAAAAAAAAACATATAATCTAAAACACC |
| Amn_1 | chr12 | + | TTTAGGTGGTGTTGGTGGAGAT | ACRAACTCTAATTCAACTCTAAATCTAC |
| Amn_2 | chr12 | + | TTATGAYGGTAGATTTAGAGTTGAATTA | RCAAATAAACCCCTAAAACAACC |
| Cacna1b_1 | chr2 | - | GGGTTAGTTAGTTAAGGTTTTAGAATT | RTACCTTCAAACTTTAACTTACTCC |
| Cacna1b_2 | chr2 | - | GTTGGGAGTAAGTTAAAGTTTGAAGG | TCAACCCCTAAAATCTCCTTACTATAAC |
| Srd5a2_1 | chr17 | - | GGGTTATAATGGGGAGGGAATTTTGGTT | TTTCCCAAAACACAAAATCAAAATCCCCAT |
| Srd5a2_2 | chr17 | - | GTATAGTTTGGTTTTTGTAGGAGTTG | AAACTCCAAACCTACCTTACTCTA |
| Cdkn2b | chr4 | - | TAGGTGGTAGAGTTGTTGTTGTTTTA | RATAACCCTACTCTTCAACCAAATCT |
| Ltbp3_1 | chr19 | + | GAGYGGGATAGTTTTGAGGTTAT | CACTCACAAACTACTTAAACAATATATCCT |
| Ltbp3_2 | chr19 | + | GTTTTTGTTAGGTGTTTGTTGTAGGAAT | TCCTAACCCCAAAAACACCAAAAA |
| Scn8a | chr15 | + | TTTTTYGATATAATAGTAAAAGTAGTATTTTTAGTTTT | CRAACRACTACACTAACTATAACC |
