# Supplementary material for: Hypermethylation of gene body CpG islands predicts high dosage of functional oncogenes in liver cancer
Source: Nat Commun. 2018 Aug 8;9:3164. doi: 10.1038/s41467-018-05550-5 (PMC6082886; doi:10.1038/s41467-018-05550-5)
Supplement: Supplementary file 13 — Supplementary Data 10 [file 41467_2018_5550_MOESM13_ESM.docx]

**Supplementary Data 10**

|  |  |  |  | **Pubmed** | |
| --- | --- | --- | --- | --- | --- |
|  |  | **r_d_to_ATG** | **RNAseq** | **Cancer** | **HCC** |
| Group I | Grb10 | -36,7634055 | 2,27775258 | 83 | 0 |
|  | Trp53inp1 | -31,097561 | 1,09798178 | 8 | 0 |
|  | Jun | -26,2329932 | 1,22301586 | 312643 | 8882 |
|  | Ptprs | -21,7980099 | 1,25122966 | 29 | 1 |
|  | Met | -19,8416633 | 2,29865478 | 23705 | 979 |
|  | Cldn7 | -9,96732026 | 2,99845412 | 131 | 5 |
|  | Mt1 | -9,41739824 | 2,93011364 | 1469 | 47 |
|  | Slc20a1 | -9,16643779 | 1,77173199 | 12 | 0 |
|  | Gata2 | -6,28364147 | 1,06117479 | 350 | 5 |
|  | Mgat4a | -4,43276935 | 1,37312893 | 3 | 3 |
|  | Tspan9 | -0,41260979 | 1,08328276 | 4 | 0 |
|  | Srgap1 | -0,17705475 | 1,74454695 | 14 | 0 |
|  | Ptk7 | -0,08310249 | 3,04149435 | 74 | 2 |
|  | Dbn1 | -0,06190247 | 1,36244806 | 6 | 0 |
|  | Prickle2 | -0,01341906 | 1,16116453 | 11 | 0 |
|  | Rundc3b | -0,0120929 | 1,81227383 | 4 | 0 |
|  | Dysf | -0,00098937 | 1,08847109 | 7 | 0 |
|  | Prrx1 | 0,13329976 | 1,58701577 | 51 | 3 |
|  | Arhgap27 | 0,23795764 | 2,69215105 | 4 | 0 |
|  | Cpeb1 | 0,84554417 | 1,85726457 | 32 | 0 |
|  | Efnb2 | 1,13025755 | 1,93891513 | 48 | 0 |
|  | Chrna4 | 1,13344867 | 2,61676334 | 9 | 0 |
|  | Pcdh17 | 1,14658224 | 2,91424797 | 21 | 0 |
|  | Srd5a2 | 1,1785406 | 2,54417107 | 466 | 10 |
|  | Bhlha15 | 4,18397626 | 1,69763893 | 6 | 0 |
|  | Inhbb | 5,81643543 | 5,64416433 | 39 | 0 |
|  | Hmgn2 | 7,84760664 | 1,0559603 | 81 | 0 |
|  | Gabbr1 | 14,7187715 | 1,07242652 | 11 | 0 |
|  | C2cd4c | 15,1440074 | 4,85856967 | 0 | 0 |
|  | Foxc1 | 20,2742124 | 4,06526851 | 100 | 6 |
|  | Ltbp3 | 21,1146575 | 2,55658057 | 12 | 4 |
| Group II | Relb | 42,1354416 | 1,66555286 | 367 | 8 |
|  | Scn8a | 43,3074242 | 6,88952195 | 11 | 0 |
|  | Ano8 | 47,3935576 | 1,20855826 | 1 | 0 |
|  | Nfkb2 | 49,0389354 | 1,62482441 | 215 | 3 |
|  | Cacna1b | 52,5367714 | 9,24109778 | 11 | 0 |
|  | Irx3 | 55,7509158 | 3,37349559 | 15 | 1 |
|  | Cdkn1c | 62,1781342 | 1,22010356 | 330 | 21 |
|  | Tbc1d2 | 64,2677183 | 1,01971009 | 3 | 0 |
|  | Tmem191c | 73,253833 | 2,0414113 | 0 | 0 |
|  | Dusp8 | 74,0924401 | 1,27195817 | 6 | 0 |
|  | Adamtsl5 | 75,9569378 | 3,21299814 | 2 | 0 |
|  | Neurl1b | 77,2324559 | 2,14294317 | 1 | 0 |
|  | Pou3f1 | 78,6101695 | 4,54956012 | 11 | 0 |
|  | Btbd17 | 78,6293436 | 5,7718442 | 1 | 1 |
|  | Cdkn2b | 81,1266356 | 6,60343493 | 844 | 33 |
|  | Map3k6 | 82,8125 | 2,3446564 | 7 | 0 |
|  | Amn | 83,9585297 | 3,55348809 | 103 | 1 |
|  | Arhgap22 | 87,7496288 | 5,44812748 | 6 | 0 |
|  | Cdkn2a | 87,9806493 | 4,98511797 | 7321 | 238 |
|  | Snx20 | 88,3840379 | 2,93018095 | 1 | 0 |
|  | Neurl1a | 92,8815255 | 3,74688888 | 0 | 0 |
|  | Hcn2 | 97,1604938 | 2,99977547 | 11 | 0 |
|  | Ssbp4 | 97,1939014 | 2,14006613 | 1 | 0 |
|  | Actn1 | 99,0902215 | 1,06043394 | 27 | 0 |
