# Supplementary material for: Hypermethylation of gene body CpG islands predicts high dosage of functional oncogenes in liver cancer
Source: Nat Commun. 2018 Aug 8;9:3164. doi: 10.1038/s41467-018-05550-5 (PMC6082886; doi:10.1038/s41467-018-05550-5)
Supplement: Supplementary file 14 — Supplementary Data 11 [file 41467_2018_5550_MOESM14_ESM.docx]

**Supplementary Data 11**

| **Gene** | **Forward (5’ >> 3’)** | **Reverse (5’ >> 3’)** |
| --- | --- | --- |
| *Tspan9* | CACAGTTCCTGACAGATGCTGC | CGTGCTTGTTGTCATCAAACCAC |
| *Arhgap27* | AGTGCTGATTTCGGGTCCAG | AAAGACTGCAGAGTGGGTCG |
| *Pcdh17* | GCTCCACGTTTAAGGACCCA | TCTGGTTCTTGTTCGAGTGGC |
| *Srd5a2* | CGGTGTCTTCTTCTTTATTCT | TAGCCCATCCATTCAATAATC |
| *Inhbb* | CAGTCTCCAGAGCGACTTGC | GATCGGGGTGAAGCGAAAGC |
| *C2cd24* | AGAACAGAATGGACCGCCTG | CTAGGTGCTTGGCTCACCTC |
| *Ltbp3* | GCTTCAAGGTGGTCTTTGCG | CGTTCATGCAGGGTAGAGGG |
| *Scn8a* | GACTTGAGGAAGAGGAACTAG | GTAAGGAAGGAGATACAGCAA |
| *NFkB2* | TGCCATTGTGTTCCGGACACC | CGCTTCCTCTGCACTTCCTCC |
| *Cacna1b* | CTTCATGGGAGGATCCAAAGG | CGGCAATGACGGCAAATATGA |
| *AdamtsL5* | TCCGTTCTTGCTGTGGACTCTC | GACCACAAGAGCTGGAACACCT |
| *Neurl1b* | GTGTCAACGATGGTGAGCCA | AAGGTGCTTTCCAGAAGCTGT |
| *Btbd17* | CCAGAGTGAGGTTATGCTGCGG | GCTGCAAGGATGCTACTCGGTA |
| *Cdkn2b* | TCCACAGGCTAAATGGGAAACC | AATCCAGGCATCAAGGCAACT |
| *Amn* | GAGATGCTGCCGTGGATCTG | TCAACGACACGATAGCTCCG |
| *Arhgap22* | AGGCCAACTACAACCTGCTC | TGAACCAGAGACGTGCCTTC |
| *Cdkn2a* | TTAGCTTCACTTCTAGCGATG | GTTTCTCATGCCATTCCTTTC |
| *Actn1* | AGATTGACCAGCTGGAGTGC | TGAACTCTTCGGGACCCAAC |
| *Hprt* | CTGGTGAAAAGGACCTCTCGAAG | CCAGTTTCACTAATGACACAAACG |
