# Supplementary material for: Hypermethylation of gene body CpG islands predicts high dosage of functional oncogenes in liver cancer
Source: Nat Commun. 2018 Aug 8;9:3164. doi: 10.1038/s41467-018-05550-5 (PMC6082886; doi:10.1038/s41467-018-05550-5)
Supplement: Supplementary file 15 — Supplementary Data 12 [file 41467_2018_5550_MOESM15_ESM.docx]

**Supplementary Data 12**

| **Genes** | **sh Number** | **References** | **Target Sequences (5’----3’)** |
| --- | --- | --- | --- |
| *Scn8a* | Sh1 | TRCN0000366185 (Sigma) | CCGGAGGACTTTGACCCGTACTATTCTCGAGAATAGTACGGGTCAAAGTCCTTTTTTG |
|  | Sh2 | TRCN0000069061  (Sigma) | CCGGGCTGGTATAAGTTTGCCAATACTCGAGTATTGGCAAACTTATACCAGCTTTTTG |
| *Actn1* | Sh1 | TRCN0000090178  (Sigma) | CCGGCCACAAAGTGACAGTTTACAACTCGAGTTGTAAACTGTCACTTTGTGGTTTTTG |
|  | Sh2 | TRCN0000090179  (Sigma) | CCGGCCAGGAACAGATGAACGAATTCTCGAGAATTCGTTCATCTGTTCCTGGTTTTTG |
| *Srd5a2* | Sh1 | TRCN0000039179  (Sigma) | CCGGCCTGGTTTATTGCGCGGAATACTCGAGTATTCCGCGCAATAAACCAGGTTTTTG |
|  | Sh2 | TRCN0000039180  (Sigma) | CCGGCGTCGGTGTCTTCTTCTTTATCTCGAGATAAAGAAGAAGACACCGACGTTTTTG |
| *NFkB2* | Sh1 | TRCN0000012344  (Sigma) | CCGGGCGAGGCTTCAGATTTCGATACTCGAGTATCGAAATCTGAAGCCTCGCTTTTT |
|  | Sh2 | TRCN0000235404  (Sigma) | CCGGCTCTCCCACAGACGTTCATAACTCGAGTTATGAACGTCTGTGGGAGAGTTTTTG |
| *Neurl1b* | Sh1 | MSH026338-21-nU6  (GeneCopoeia) | GAAGTACAGCTTCTGGAAAGC |
|  | Sh2 | MSH026338-22-nU6 (GeneCopoeia) | GCCAACTTCGACAACAACGAG |
